# Impact of Inter-fractional Anatomical Changes on Dose Distributions in Passive Carbon-Ion Radiotherapy for Prostate Cancer: Comparison of Vertical and Horizontal Fields

**DOI:** 10.3389/fonc.2020.01264

**Published:** 2020-07-28

**Authors:** Ayaka Yokoyama, Yoshiki Kubota, Hidemasa Kawamura, Yuhei Miyasaka, Nobuteru Kubo, Hiro Sato, Satoshi Abe, Kazuhisa Tsuda, Takayuki Sutou, Tatsuya Ohno, Takashi Nakano

**Affiliations:** ^1^Department of Radiology, Gunma University Hospital, Maebashi, Japan; ^2^Gunma University Heavy Ion Medical Center, Maebashi, Japan

**Keywords:** carbon-ion radiotherapy, prostate cancer, patient positioning, inter-fractional anatomical change, adequate margin

## Abstract

**Purpose:** We quantified the inter-fractional changes associated with passive carbon-ion radiotherapy using vertical and horizontal beam fields for prostate cancer.

**Methods:** In total, 118 treatment-room computed tomography (TRCT) image sets were acquired from 10 patients. Vertical (anterior–posterior) and horizontal (left–right) fields were generated on the planning target volume identified by treatment planning CT. The dose distribution for each field was recalculated on each TRCT image set at the bone-matching position and evaluated using the dose–volume parameters for the prostate and rectum V95 values. To confirm adequate margins, we generated vertical and horizontal fields with 0-, 2-, 4-, and 6-mm isotropic margins from the prostate and recalculated the dose distributions on all TRCT image sets. Sigmoid functions were fitted to a plot of acceptable ratios (that is, when prostate V95 > 98%) vs. the isotropic margin size to identify the margin at which this ratio was achieved in 95% of patients with a vertical or horizontal field.

**Results:** The prostate V95 values (mean ± standard deviation) were 99.89 ± 0.62% and 99.99 ± 0.00% with vertical and horizontal fields, respectively; this difference was not statistically significant (*p* = 0.067). The rectum V95 values were 1.93 ± 1.25 and 1.88 ± 0.96 ml with vertical and horizontal fields, respectively; the difference was not statistically significant (*p* = 0.432). The estimated adequate margins were 2.2 and 3.0 mm for vertical and horizontal fields, respectively.

**Conclusions:** Although there is no significant difference, horizontal fields offer higher reproducibility for prostate dosing than vertical fields in our clinical setting, and 3.0 mm was found to be an adequate margin for inter-fractional changes.

## Introduction

Prostate cancer is the second most common cancer in males according to the International Agency for Research on Cancer (1). The outcomes of radiotherapy are equal or better than those of surgery (2). One type of radiotherapy, carbon-ion radiotherapy (CIRT), reportedly reduces the risk of acute and late toxicities with outcomes that are equal or better than those of conformal radiotherapy and intensity-modulated radiation therapy (3–7).

Carbon-ion beams provide sharper dose distributions than photon beams because they benefit from the Blagg peak and a sharp lateral penumbra (8, 9). However, carbon-ion beams are sensitive to changes in the target position or water-equivalent path length (WEL) to the target, which may result in changes in dose distributions (10–14). The reproducibility of dose distributions for inter-fractional anatomical changes is very important to ensure safe treatment of patients; however, few reports have focused on this topic in the context CIRT for prostate cancer.

In our previous study, we evaluated the influence of the beam field angle during setup the range uncertainty on the rectal and target doses in CIRT for prostate cancer (15). Our results showed that the prostate and rectal dose deviations did not vary significantly with the field angle. However, while the setup uncertainty was considered in this study, inter-fractional anatomical changes in the prostate, bladder, and/or rectum were not. To improve the safety of CIRT, it is necessary to evaluate the influence of such anatomical changes on the dose distribution. Additionally, whether a vertical or horizontal field is more robust against inter-fractional changes remains unclear. Further, because a vertical field must be used instead of a horizontal field in certain cases, such as when the patient has a metal hip implant, it is important to evaluate the robustness of a vertical field against inter-fractional changes. Hence, in this study, we evaluated the robustness of horizontal and vertical fields against inter-fractional anatomical changes using daily computed tomography (CT) images acquired in a treatment room.

## Materials and Methods

### Patient

This prospective study included 10 consecutive patients with prostate cancer who had agreed to participate in this study and had been treated with 12 fractions of passive-irradiation CIRT at Gunma University Heavy Ion Medical Center from June 2017 to March 2018. The patients' characteristics are detailed in Table 1. This study was conducted in accordance with the Declaration of Helsinki and approved by the institutional review board at Gunma University Hospital (1564). The study was registered at the University Hospital Medical Information Network Clinical Trials Registry (UMIN-CTR trial number: 000029495). All patients provided written informed consent to participate in this study and their data were anonymized.

**Table 1:** Patient characteristics.

**Patient Number**	**Age**	**Prostate Volume (ml)**	**Rectal Volume (ml)**	**Bladder Volume (ml)**
P1	67	17.2	48.5	322.5
P2	72	18.7	70.5	192.8
P3	59	22.0	68.9	198.5
P4	76	17.4	38.7	214.2
P5	73	23.4	44.6	139.1
P6	76	20.6	48.0	146.3
P7	66	15.0	31.7	155.4
P8	70	18.5	39.8	113.3
P9	60	32.8	35.5	89.7
P10	78	19.2	61.1	105.2
Median	71	18.95	46.3	150.85

*The prostate, rectal, and bladder volumes were measured from the treatment planning CT*.

### CT Image Acquisition and Actual Treatment

Twelve CT data sets were acquired on each day of treatment to investigate the effects of tumor movement and inter-fractional changes on the dose decided in the treatment planning stage. All patients were immobilized in the supine position by a shell fitter (Kuraray, Tokyo, Japan) to depress the abdomen and prevent body movement. A MoldCare cushion (Alcare, Tokyo, Japan) was used to provide trunk support while the patient was irradiated and CT images were acquired. CT images for treatment planning (PlanCT) were acquired on a scanner (Aquilion LB®, Self-Propelled; Canon Medical Systems, Otawara, Tochigi, Japan) in a simulation room.

Each patient retained his urine for 20 min before entering the irradiation room. The patient was positioned with the aid of orthogonal X-ray imaging (16, 17). If gas or feces was observed inside or close to the target on the X-ray images, a degassing or enema procedure was performed. This radiotherapy irradiation procedure was performed on each of the 12 separate days. After the radiotherapy, one set of CT images was acquired using a treatment-room CT (TRCT) system of the same type as in the simulation room (18). TRCT image sets were obtained from each patient in the same position as used for irradiation and with the same tube voltage, tube current, field of view, and slice thickness settings used for the PlanCT.

### Treatment Planning

In this study, we used a CIRT system (19) with a heavy ion irradiation device (Mitsubishi Electric, Tokyo, Japan) with passive irradiation (20) and a treatment planning system (TPS) (XiO-N, Mitsubishi Electric). The passive irradiation field was generated using a scatterer and wobbling, and the field was collimated to the outside of the planning target volume (PTV) using a multi-leaf collimator. A pencil-beam algorithm was used to calculate the dose distributions (21, 22). The relative biological effectiveness (RBE) was included in the absorbed dose using a spread-out Bragg peak concept (23), and the clinical dose was defined as Gy (RBE). The PTV1 for the prostate cancer treatment for each patient was determined after adding 8-mm anterior and lateral margins, 6-mm cranial and caudal margins, and a 5-mm posterior margin to the prostate as well as 3-mm lateral margins, 5-mm cranial and caudal margins, and a 5-mm posterior margin to the proximal seminal vesicle. The PTV2 was created by subtracting 6 mm from the circumscribed position of the PTV1 in the cranial and caudal directions and subtracting the circumscribed region of the rectum in the posterior direction of PTV1 from the PTV1. The CIRT plan was generated such that the percentages of PTV1 and PTV2 receiving >95% of the prescribed dose (V95) were >95%. Irradiation was applied with fields from the left and right sides. Two of the fields were applied in four fractions, and the other two were applied in two fractions, which results in an initial field of 8 fractions to PTV1 and a boost field of 4 fractions to PTV2; thus, there was a total of 12 fractions. Each fraction was 4.3 Gy (RBE), and the total dose was therefore 4.3 × 12 = 51.6 Gy (RBE).

### Data Analysis

The inter-fractional prostate displacements were measured from bone-matching positions to prostate-matching positions between the PlanCT and subsequent TRCT images for each patient (*n* = 118; 2 CT sets were not acquired because of a CT system failure) using commercial software (MIM Maestro® MIM Software, Cleveland, OH, USA). The registration was based on the translation in three directions (left–right (LR), anterior–posterior (AP), and superior–inferior (SI), each defined as + and – values) because CT images cannot be rotated for dose calculation with the XiO-N system. Prostate contours were generated on all TRCT images by the rigid image registration method based on the PlanCT, and the rectum and bladder were manually delineated on all TRCT images. After generation and delineation, an oncologist and medical physicist checked the contours. Additionally, deviations from the volumes on the PlanCT to those measured on each TRCT were calculated. The WELs in the AP direction were then measured from the patient's body surface to the isocenter of the beam's direction plane, and the correlation between the prostate displacements and the WEL deviations in the AP direction was evaluated.

**Figure 1:**
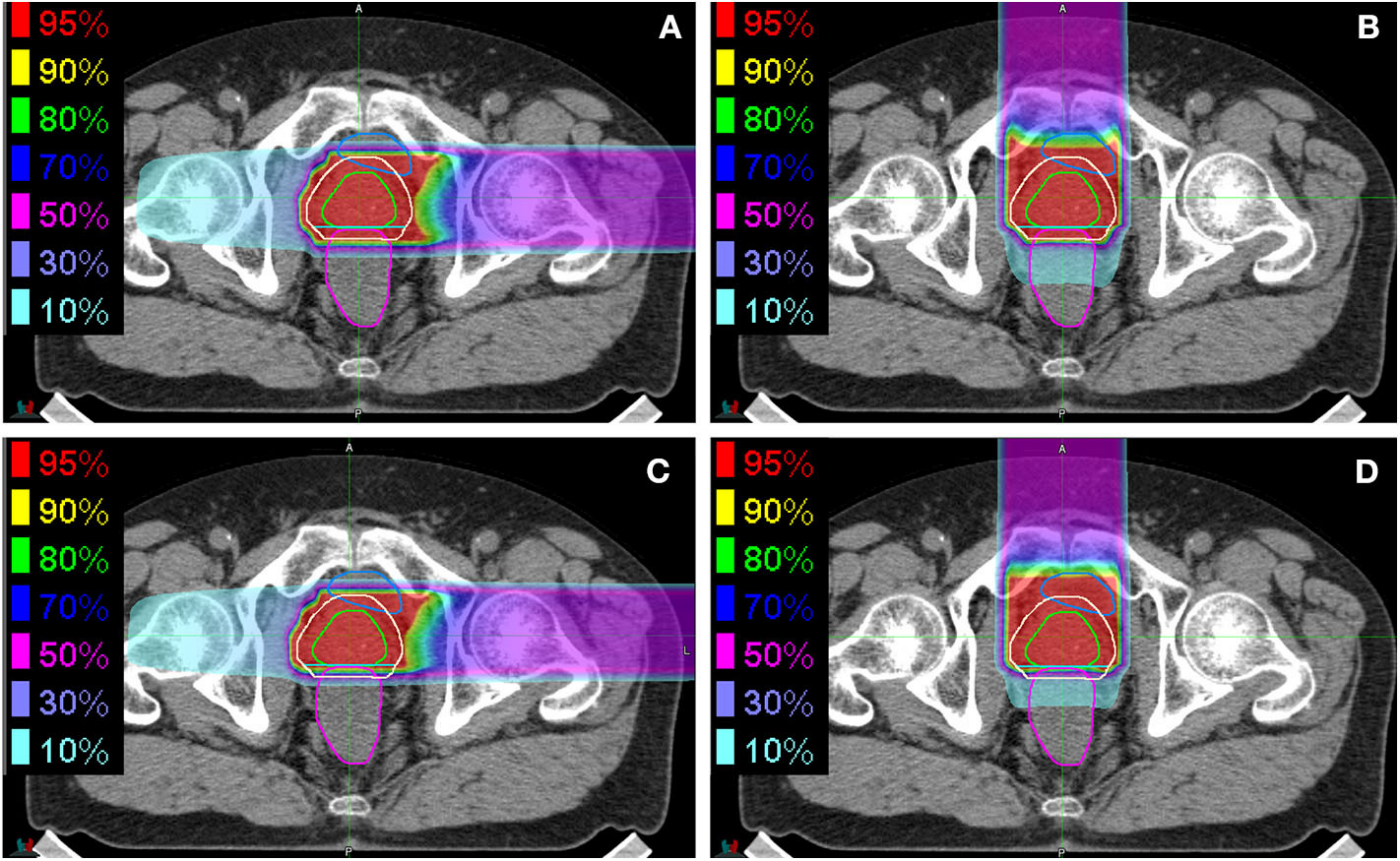
Examples of dose distributions for **(A,B)** initial fields and **(C,D)** boost fields from **(A,C)** the horizontal direction and **(B,D)** the vertical direction. The green, almond, cyan, blue, and magenta lines delineate the prostate, PTV1, PTV2, bladder, and rectum, respectively.

Initial and boost fields on horizontal and vertical (LR and AP directions, respectively) were generated and used for evaluation. Examples of the dose distributions associated with vertical and horizontal fields are shown in Figure 1. The daily dose distributions for the initial and boost fields in the vertical and horizontal directions were recalculated on all TRCT sets at the bone-matching position. The dose–volume parameters of the prostate V95, rectum V95, V50, and V10 were also evaluated. For the rectal volume evaluation, the rectal wall was considered to be 3 mm thick, as described previously (24, 25). Additionally, the correlation between the prostate displacement and the dose–volume parameters of the prostate V95 and those between the rectal volume deviation and the deviations in the dose–volume parameters of the rectum V95, V50, and V10 were evaluated.

To estimate the appropriate margins in CIRT for prostate cancer to ensure robustness against inter-fractional anatomical changes, vertical and horizontal fields were generated on the PlanCT with 0-, 2-, 4-, and 6-mm isotropic margins to the prostate. The dose distributions were then recalculated for all TRCT images at the bone-matching position. Sigmoid functions were fitted to the plot of the acceptance ratio vs. the isotropic margin size to identify the margin that enables 95% of the examined patients to achieve an acceptable condition (prostate V95 > 98%) for each field in the vertical and horizontal directions.

### Statistics

All dose–volume parameters for vertical and horizontal fields, as well as the prostate displacements and WEL deviations in the AP direction, were compared using *t*-tests; *p* = 0.05 was considered statistically significant.

## Results

**Figure 2:**
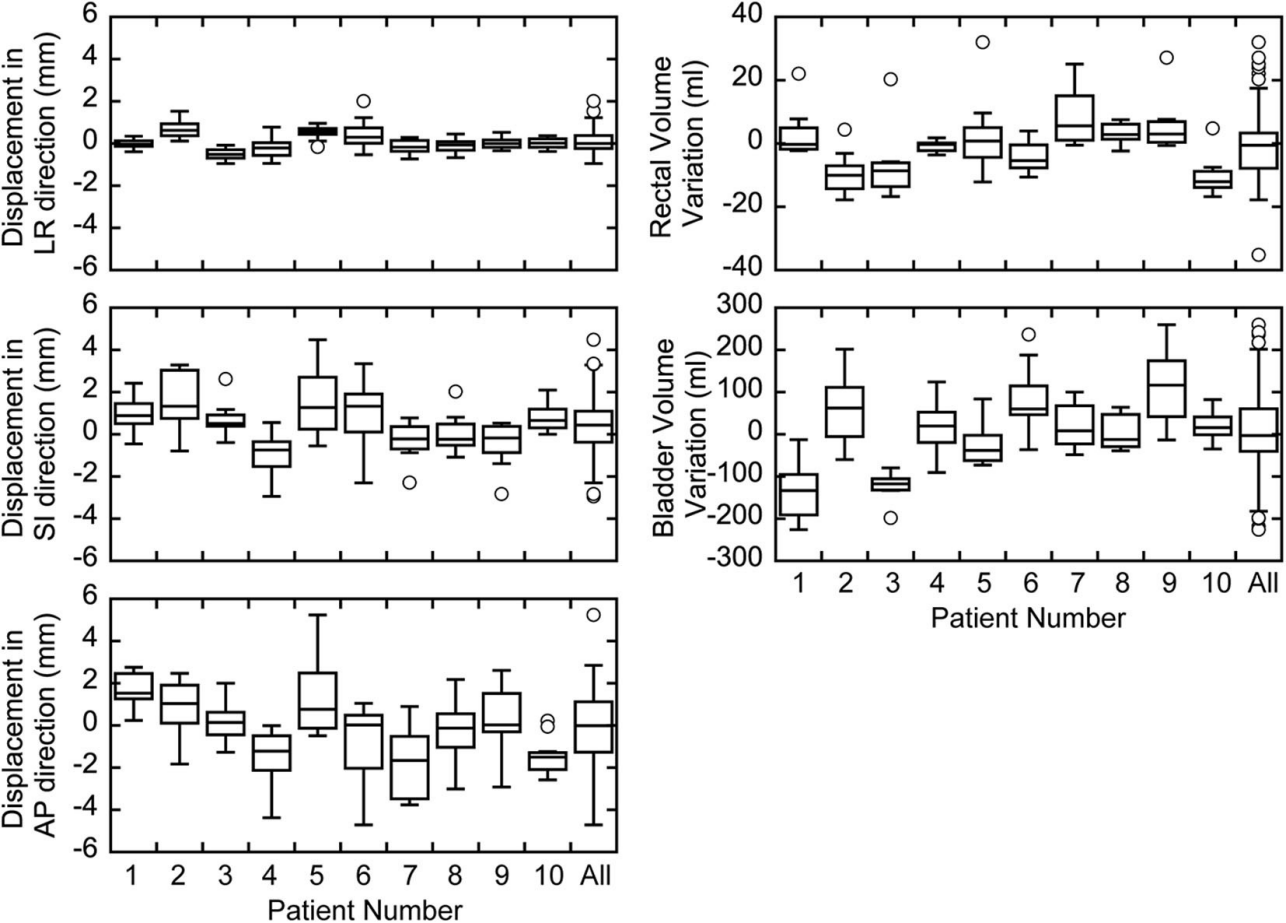
Box plots of prostate displacements and inter-fractional variations in the rectal and bladder volumes for each patient.

**Figure 3:**
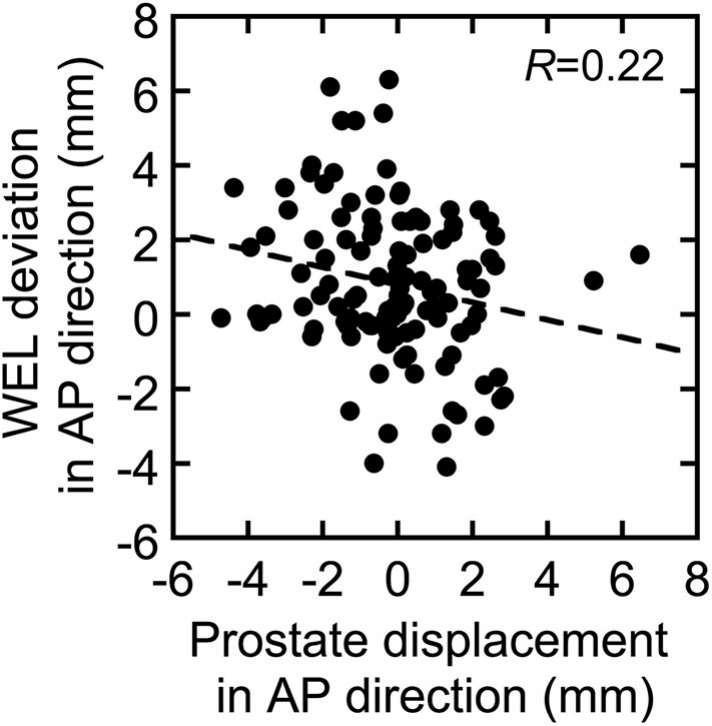
Correlation between prostate displacement and water-equivalent path length (WEL) deviation in the AP direction. The dotted line shows linear fitting to the data.

The measured prostate displacements and rectal and bladder volume variations are shown in Figure 2. The mean ± standard deviation of the prostate displacement for all patients were 0.08 ± 0.50, 0.46 ± 1.32, and −0.12 ± 1.87 mm in the LR, SI, and AP directions, respectively, and those for the rectal and bladder volume deviations were −1.07 ± 9.37 and 2.55 ± 95.36 ml, respectively. The correlation between prostate displacement and WEL deviation in the AP direction is shown in Figure 3. The mean ± standard deviation of the prostate displacement and WEL deviation were −0.13 ± 1.88 and 0.82 ± 2.04 mm, respectively; the mean difference was not statistically significant.

The dose–volume parameters on PlanCT and TRCT are shown in Table 2. There were no statistically significant differences between the daily prostate V95 with initial fields in the vertical and horizontal directions. For the boost field, however, the prostate V95 was significantly lower in the vertical than horizontal direction.

**Table 2:** Dose volume of prostate and rectum.

			**Initial**			**Boost**		
			**Vertical**	**Horizontal**	***p*-value**	**Vertical**	**Horizontal**	***p*-value**
Plan	Prostate	V95 (%)	100 ± 0	100 ± 0	–	99.89 ± 0.07	99.99 ± 0.02	0.002
	Rectum	V95 (ml)	2.03 ± 0.48	1.93 ± 0.38	0.097	0.01 ± 0.02	0.03 ± 0.04	0.188
		V50 (ml)	3.10 ± 0.64	3.64 ± 0.70	<0.001	1.06 ± 0.23	1.90 ± 0.40	<0.001
		V10 (ml)	9.42 ± 1.79	5.11 ± 0.90	<0.001	6.08 ± 0.94	3.26 ± 0.67	<0.001
Daily	Prostate	V95 (%)	99.89 ± 0.62	100.00 ± 0.00	0.067	95.95 ± 5.81	97.88 ± 3.87	<0.001
	Rectum	V95 (ml)	1.93 ± 1.25	1.88 ± 0.96	0.432	0.37 ± 0.69	0.43 ± 0.65	0.145
		V50 (ml)	3.09 ± 1.63	3.57 ± 1.14	<0.001	1.19 ± 1.28	1.83 ± 0.98	<0.001
		V10 (ml)	9.43 ± 2.16	5.04 ± 1.37	<0.001	6.06 ± 1.99	3.22 ± 1.11	<0.001
Deviation	Prostate	V95 (%)	−0.11 ± 0.62	0.00 ± 0.00	0.067	−4.06 ± 5.83	−2.12 ± 3.88	<0.001
	Rectum	V95 (ml)	−0.10 ± 1.22	−0.05 ± 1.00	0.349	0.36 ± 0.69	0.40 ± 0.66	0.317
		V50 (ml)	−0.01 ± 1.60	−0.07 ± 1.14	0.402	0.14 ± 1.30	−0.07 ± 1.00	0.005
		V10 (ml)	0.03 ± 1.80	−0.07 ± 1.34	0.258	−0.01 ± 1.67	−0.03 ± 1.17	0.881

*The Plan values show the mean ± standard deviation for each of 10 patients, the Daily values show the mean ± standard deviation of the 118 images from irradiation days, and the Deviation values show the mean ± standard deviations of the differences between the Plan and Daily values. Initial shows the fields to the PTV1, and Boost shows the field to the PTV2. Vx, volume receiving greater than x% of the prescription dose*.

**Figure 4:**
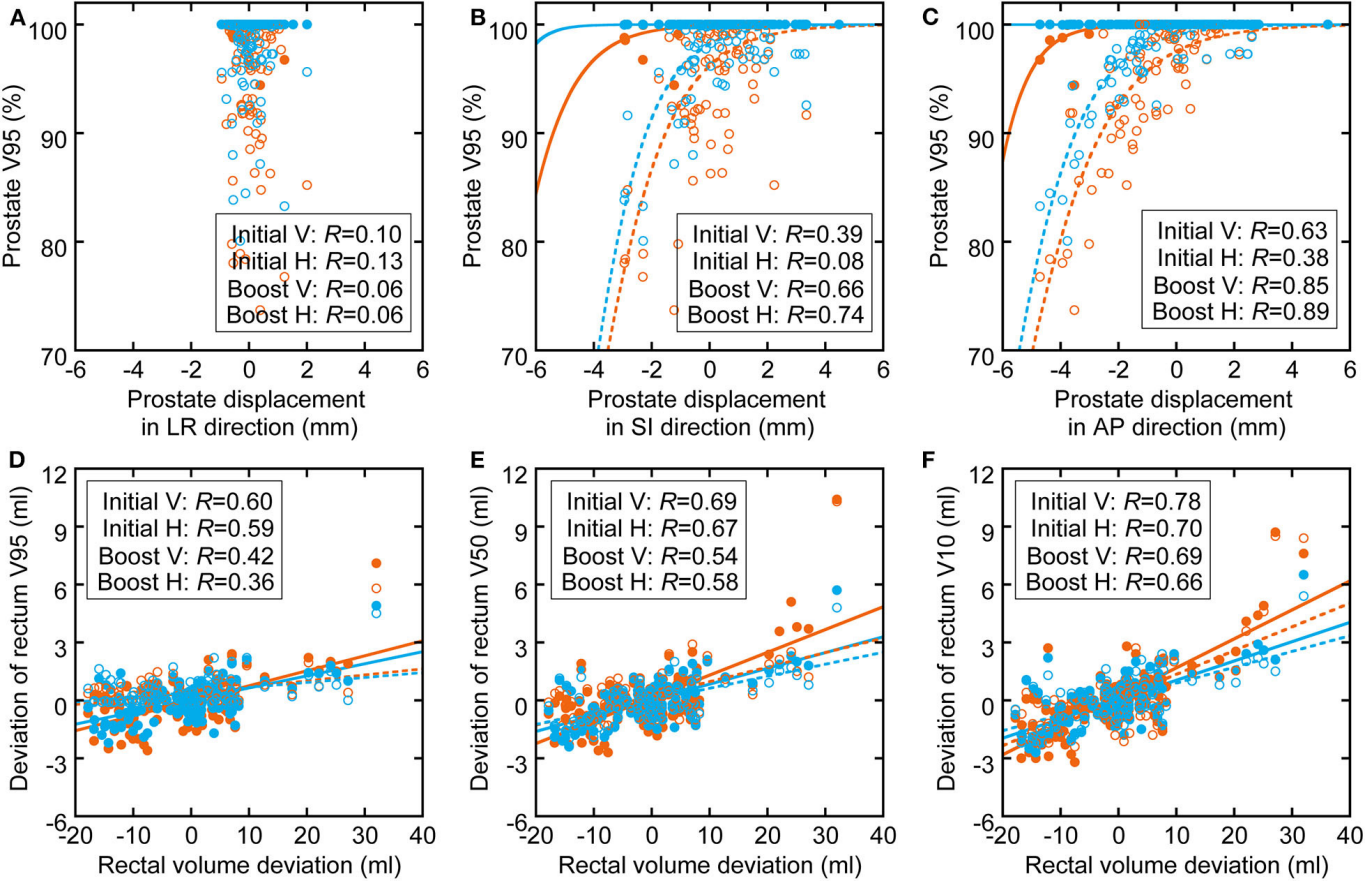
Correlation between **(A–C)** prostate displacement and prostate dose volume deviation (sigmoid fitting) and between **(D–F)** rectal volume deviation and rectal dose volume deviation (linear fitting). The orange circles and lines show the parameters of vertical fields, and the light blue circles and lines show the parameters of horizontal fields. The filled circles and solid lines show initial fields, and the hollow circles and dotted lines show boost fields.

**Figure 5:**
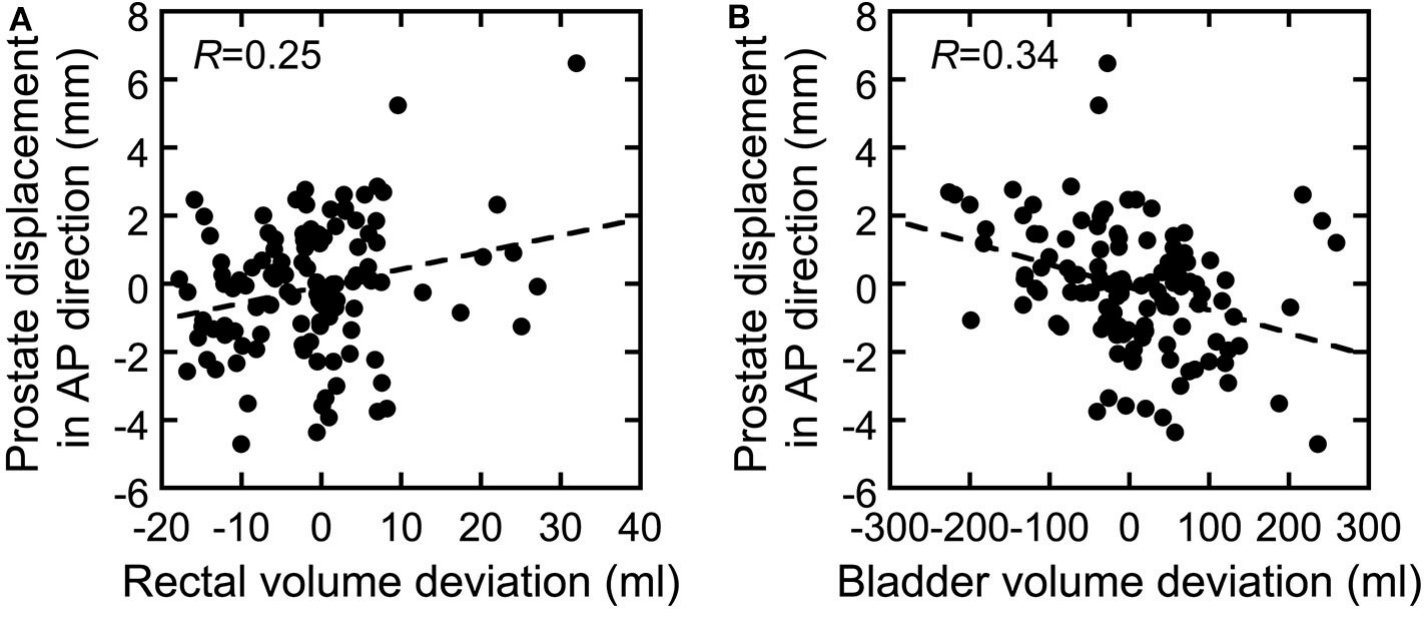
Correlation between deviations in **(A)** rectal volume and **(B)** bladder volume vs. the prostate displacement in the AP direction.

**Figure 6:**
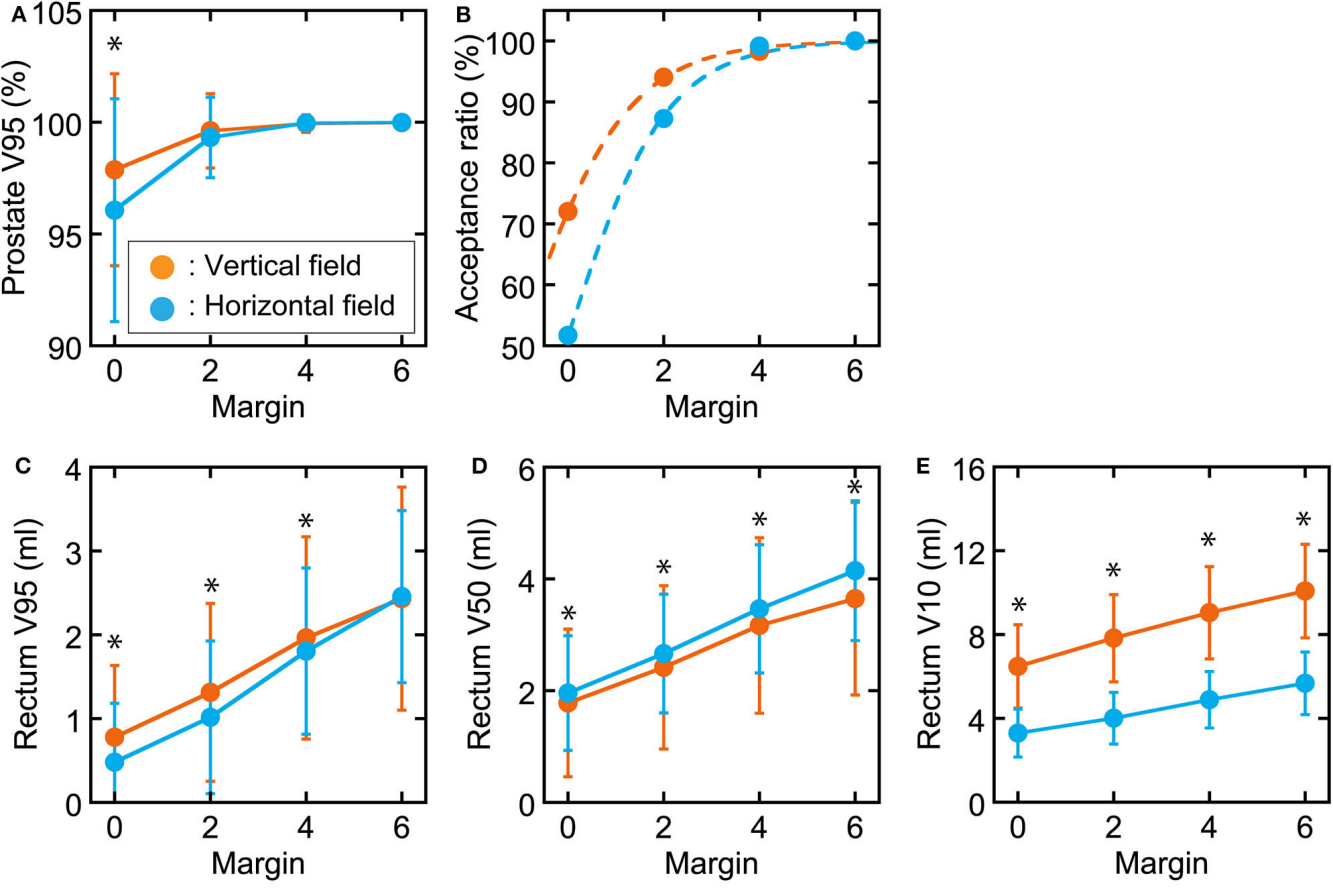
Graphs of prostate and rectum dose–volume parameters and acceptance ratio in each margin. **(A)** Prostate V95. **(B)** Acceptance ratio when prostate V95 > 98% with fitted sigmoid functions. Rectal **(C)** V95, **(D)** V50, and **(E)** V10 values as functions of the margin used. Orange circles show vertical fields, and light blue circles show horizontal fields. *Statistically significant difference.

The correlations between prostate displacement and prostate V95 and between rectal volume deviation and rectal dose volume are shown in Figure 4. The correlations of prostate displacement with bladder volume deviation and rectal volume deviation in the AP direction are shown in Figure 5. The prostate and rectal dose volume and acceptance ratio graphs are shown in Figure 6. Based on these data, adequate margins in the vertical field and horizontal field for an acceptance ratio of 95% were determined to be 2.2 and 3.0 mm, respectively.

## Discussion

Our results showed that the average inter-fractional prostate displacement was 0.46 ± 1.32 mm in the SI direction and −0.12 ± 1.87 mm in the AP direction. Our measured displacements were similar to those measured from in-room CT (26) using a flat-type shell similar to that used in this study. However, these displacements were smaller than those measured under cone-beam CT (27, 28) or megavoltage CT (29) with different shell types or without the shell. It was hypothesized that the patient immobilization induced by pressing with a flat shell may also suppress prostate displacement.

Table 2 shows that the prostate coverage was better with the horizontal field than with the vertical field. The difference was statistically significant for the boost fields because they do not have a margin in the posterior direction from the prostate, which results in less consistent coverage than with the initial fields, which have a large margin. There are two possible reasons for the target coverage with the vertical field being worse than that with the horizontal field. The first is that the coverage of the vertical field in the treatment planning is slightly worse than that of the horizontal field. Because the dose calculation in XiO-N is a forward calculation, the 95% isodose line does not perfectly match the PTV. This effect is mostly observed along the beam axis rather than in the lateral direction perpendicular to the beam and results in worse coverage on the posterior side of the prostate with the vertical field than with the horizontal field. The second possible explanation is that the prostate displacements in the AP direction are slightly larger than the WEL changes in the AP direction, as illustrated in Figure 3, although the difference was not statistically significant. The prostate displacements in the AP direction affect the dose distribution in the vertical field, while the WEL deviations in the AP direction influence the dose distribution in the horizontal field. Thus, it is possible that the vertical field is more strongly affected by inter-fractional WEL deviations than is the horizontal field by the prostate displacements, which results in worse target coverage with the vertical field. Hence, if target coverage is a priority, the use of a vertical boost field may not be ideal.

There was no significant difference in the rectal wall dose between the rectum V95 values associated with the initial and boost fields. However, the vertical fields resulted in significantly lower rectum V50 values and significantly higher V10 values than the horizontal fields. It was assumed that the distal fall off in the vertical field was steeper than the lateral penumbra in the horizontal field, which explains why a significant difference was observed in the rectum V50 but not in the V95. Additionally, the difference in the rectum V10 values was attributed to the fact that the dose on the distal tail in the vertical field was higher than that on the lateral tail in the horizontal field. While these results capture inter-fractional anatomical changes, similar tendencies were observed in our previous study considering setup uncertainties and beam range uncertainties (15). Therefore, it can be concluded that a vertical field is more effective for reducing the rectal middle dose, while a horizontal field is more effective for reducing the rectal low dose.

Figure 4 shows that the correlation coefficients between the inter-fractional prostate displacement and prostate coverage were high in the SI and AP directions but low in the LR direction. Furthermore, the correlations of the prostate displacement with the deviations in the bladder volume and rectal volume were low (*R* = 0.25 and 0.34), as shown in Figure 5. This finding indicates that it is difficult to control the prostate displacement only by managing the inter-fractional bladder volume, rectal gas, and presence of feces in the rectum. However, monitoring the bladder volume and rectal gas and feces may effectively prevent changes in the bladder and rectal volumes as shown in this study because the TRCT images were acquired after these steps were taken; hence, such management techniques may be necessary to ensure patient safety. Because vertical fields are more sensitive to prostate displacements (because the fitting curve is steeper than in the horizontal field), and because the correlation coefficients between the rectal volume deviation and rectal wall dose volume ranged from medium to high, it can be inferred that managing the rectal gas or feces is important to control the rectal dose. In particular, because the ratio of the increase in the rectal dose to the increase in the rectal volume is higher with a vertical than horizontal field, more care must be taken when using a vertical field.

Figure 6 shows that vertical fields need smaller margins than horizontal fields. When generating the treatment planning beam, we use a spread-out Bragg peak size of 5 mm for a horizontal field and 10 mm for a vertical field in our facility. Therefore, an extra dose is delivered upstream of the target to ensure the target dose in each directional field. Because the inter-fractional prostate displacements tend to be larger in the AP than LR direction, as shown in Figure 2, it is assumed that vertical field, that extra dose is delivered in anterior direction, provide greater target coverage than the horizontal field when the margin is small. In practice, horizontal fields provide greater target coverage when the margin is sufficient (4 mm). Therefore, the challenge in delivering an extra dose is a situation specific to our facility; however, the same problem would occur at other facilities that use passive irradiation. Thus, 3-mm margins would be required for both vertical and horizontal fields.

The scope of this study is limited to the effects of inter-fractional changes because the TRCT images were acquired only one time after each irradiation. However, there may be more intra-fractional changes during the treatment. Although previous studies have indicated that the intra-fractional changes are smaller than the inter-fractional changes (28–30), it is necessary to consider both changes when determining the appropriate margins. Assuming that the intra-fractional change is equivalent to the inter-fractional change, a margin of 3 × √2 = 4.2 mm would be required to ensure patient safety.

Furthermore, this study focused on single beams. If using a combination of vertical and horizontal fields, the lower rectal dose would increase more than when using only a horizontal field; however, the dose distributions can be expected to be more robust because the uncertainty of each field is distributed. Additionally, this study considered only the daily dose distribution. Because accumulating dose distributions are effective for predicting treatment outcomes and toxicities (31), we plan to evaluate the cumulative dose distributions in a future study. However, care should be taken because the use of a deformable image registration method to calculate the accumulated dose may produce some errors (32).

Another limitation of this study was that although 118 TRCT images were acquired, the number of patients in the sample set was low (10 patients). Therefore, further analyses with more patient data are necessary. Additionally, our evaluation did not include the seminal vesicle volume. Because the dose coverage would decrease because of inter-fractional movements of the seminal vesicles, further evaluations are needed. Moreover, the prostate contours observed on TRCT were generated by the rigid image registration method from the PlanCT, which may include small errors because of inter-fractional anatomical changes.

## Conclusion

In this study, we evaluated the robustness of horizontal and vertical fields against inter-fractional anatomical changes using daily CT images acquired in the treatment room during CIRT for prostate cancer. The results showed that horizontal fields better ensure that the target dose is delivered than vertical fields. Vertical fields are effective for reducing the rectal middle dose, and horizontal fields are effective for reducing the rectal low dose. Finally, a 3-mm margin was found to be sufficient to ensure robustness against inter-fractional changes.

## Data Availability Statement

The datasets generated for this study are available on request to the corresponding author.

## Ethics Statement

The studies involving human participants were reviewed and approved by Institutional review board at Gunma University Hospital. The patients/participants provided their written informed consent to participate in this study. Written informed consent was obtained from the individual(s) for the publication of any potentially identifiable images or data included in this article.

## Author Contributions

AY, YK, and HK designed and directed the analyses. AY, YK, HK, YM, NK, HS, SA, and KT generated a database and performed data collection. AY, YK, and HK participated substantially in the preparation of the manuscript. TS, TO, and TN supervised the project. All authors contributed to the article and approved the submitted version.

## Conflict of Interest

The authors declare that the research was conducted in the absence of any commercial or financial relationships that could be construed as a potential conflict of interest.

## References

[1] International Agency for Research on Cancer Global Cancer Observatory (GCO). Available online at: http://gco.iarc.fr/ (accessed January 20, 2020).

[2] Hernandez DJ Nielsen ME Han M Partin AW. Contemporary evaluation of the D'amico risk classification of prostate cancer. Urology. (2007) 70:931–5. 10.1016/j.urology.2007.08.055 18068450

[3] Michalski J Winter K Roach M Markoe A Sandler HM Ryu J. Clinical outcome of patients treated with 3D conformal radiation therapy (3D-CRT) for prostate cancer on RTOG 9406. Int J Radiat Oncol Biol Phys. (2012) 83:e363–70. 10.1016/j.ijrobp.2011.12.070 22633552 PMC3361689

[4] Cahlon O Zelefsky MJ Shippy A Chan H Fuks Z Yamada Y. Ultra-high dose (86.4 Gy) IMRT for localized prostate cancer: toxicity and biochemical outcomes. Int J Radiat Oncol Biol Phys. (2008) 71:330–7. 10.1016/j.ijrobp.2007.10.004 18164858

[5] Sveistrup J af Rosenschöld PM Deasy JO Oh JH Pommer T Petersen PM. Improvement in toxicity in high risk prostate cancer patients treated with image-guided intensity-modulated radiotherapy compared to 3D conformal radiotherapy without daily image guidance. Radiat Oncol. (2014) 9:44. 10.1186/1748-717X-9-44 24495815 PMC3922544

[6] Nomiya T Tsuji H Kawamura H Ohno T Toyama S Shioyama Y. A multi-institutional analysis of prospective studies of carbon ion radiotherapy for prostate cancer: a report from the Japan Carbon ion Radiation Oncology Study Group (J-CROS). Radiother Oncol. (2016) 121:288–93. 10.1016/j.radonc.2016.10.009 27836119

[7] Kawamura H Kubo N Sato H Mizukami T Katoh H Ishikawa H. Moderately hypofractionated carbon ion radiotherapy for prostate cancer; a prospective observational study “GUNMA0702”. BMC Cancer. (2020) 20:75. 10.1186/s12885-020-6570-8 32000716 PMC6990498

[8] Kraft G Tumor therapy with heavy charged particles. Prog Part Nucl Phys. (2000) 45:S473–544. 10.1016/S0146-6410(00)00112-5

[9] Ohno T. Particle radiotherapy with carbon ion beams. EPMA J. (2013) 4:1–7. 10.1186/1878-5085-4-9 23497542 PMC3598788

[10] Irie D Saitoh JI Shirai K Abe T Kubota Y. Verification of dose distribution in carbon ion radiation therapy for stage I lung cancer. Int J Radiat Oncol Biol Phys. (2016) 96:1117–23. 10.1016/j.ijrobp.2016.09.002 27869084

[11] Sakai M Kubota Y Saitoh JI Irie D Shirai K Okada R. Robustness of patient positioning for interfractional error in carbon ion radiotherapy for stage I lung cancer: bone matching versus tumor matching. Radiother Oncol. (2018) 129:95–100. 10.1016/j.radonc.2017.10.003 29100701

[12] Abe S Kubota Y Shibuya K Koyama Y Abe T Ohno T. Fiducial marker matching versus vertebral body matching: dosimetric impact of patient positioning in carbon ion radiotherapy for primary hepatic cancer. Phys Med. (2017) 33:114–20. 10.1016/j.ejmp.2016.12.018 28057427

[13] Houweling AC Fukata K Kubota Y Shimada H Rasch CR Ohno T. The impact of interfractional anatomical changes on the accumulated dose in carbon ion therapy of pancreatic cancer patients. Radiother Oncol. (2016) 119:319–25. 10.1016/j.radonc.2016.03.004 26993417

[14] Kubota Y Katoh H Shibuya K Shiba S Abe S Sakai M. Comparison between bone matching and marker matching for evaluation of intra- and inter-fractional changes in accumulated dose of carbon ion radiotherapy for hepatocellular carcinoma. Radiother Oncol. (2019) 137:77–82. 10.1016/j.radonc.2019.04.026 31078014

[15] Kubota Y Kawamura H Sakai M Tsumuraya R Tashiro M Yusa K. Changes in rectal dose due to alterations in beam angles for setup uncertainty and range uncertainty in carbon-ion radiotherapy for prostate cancer. PLoS ONE. (2016) 11:e0153894. 10.1371/journal.pone.0153894 27097041 PMC4838308

[16] Kubota Y Tashiro M Shinohara A Abe S Souda S Okada R. Development of an automatic evaluation method for patient positioning error. J Appl Clin Med Phys. (2015) 16:100–11. 10.1120/jacmp.v16i4.5400 26219004 PMC5690021

[17] Kubota Y Hayashi H Abe S Souda S Okada R Ishii T. Evaluation of the accuracy and clinical practicality of a calculation system for patient positional displacement in carbon ion radiotherapy at five sites. J Appl Clin Med Phys. (2018) 19:144–53. 10.1002/acm2.12261 29369463 PMC5849861

[18] Li Y Kubota Y Tashiro M Ohno T. Value of three-dimensional imaging systems for image-guided carbon ion radiotherapy. Cancers. (2019) 11:297. 10.3390/cancers11030297 30832346 PMC6468538

[19] Ohno T Kanai T Yamada S Yusa K Tashiro M Shimada H. Carbon ion radiotherapy at the Gunma University heavy ion medical center: new facility set-up. Cancers. (2011) 3:4046–60. 10.3390/cancers3044046 24213124 PMC3763409

[20] Renner TR Chu WT. Wobbler facility for biomedical experiments. Med Phys. (1987) 14:825–34. 10.1118/1.596009 3683312

[21] Kanematsu N Yonai S Ishizaki A. The grid-dose-spreading algorithm for dose distribution calculation in heavy charged particle radiotherapy. Med Phys. (2008) 35:602–7. 10.1118/1.2829878 18383681

[22] Kanematsu N. Dose calculation algorithm of fast fine-heterogeneity correction for heavy charged particle radiotherapy. Phys Med. (2011) 27:97–102. 10.1016/j.ejmp.2010.05.001 20579913

[23] Kanai T Endo M Minohara S Miyahara N Koyama-ito H Matsufuji N. Biophysical characteristics of HIMAC clinical irradiation system for heavy-ion radiation therapy. Int J Radiat Oncol Biol Phys. (1999) 44:201–10. 10.1016/S0360-3016(98)00544-6 10219815

[24] Tucker SL Dong L Cheung R Johnson J Mohan R Huang EH. Comparison of rectal dose-wall histogram versus dose-volume histogram for modeling the incidence of late rectal bleeding after radiotherapy. Int J Radiat Oncol Biol Phys. (2004) 60:1589–601. 10.1016/j.ijrobp.2004.07.712 15590191

[25] Andrzejewski P Kuess P Knäusl B Pinker K Georg P Knoth J Feasibility of dominant intraprostatic lesion boosting using advanced photon-, proton- or brachytherapy. Radiother Oncol. (2015) 117:509–14. 10.1016/j.radonc.2015.07.028 26349588

[26] Maeda Y Sato Y Shibata S Bou S Yamamoto K Tamamura H. Effects of organ motion on proton prostate treatments, as determined from analysis of daily CT imaging for patient positioning. Med Phys. (2018) 45:1844–56. 10.1002/mp.12869 29574901

[27] Palombarini M Mengoli S Fantazzini P Cadioli C Degli Esposti C Frezza GP. Analysis of inter-fraction setup errors and organ motion by daily kilovoltage cone beam computed tomography in intensity modulated radiotherapy of prostate cancer. Radiat Oncol. (2012) 7:56. 10.1186/1748-717X-7-56 22472040 PMC3359229

[28] Tanyi JA He T Summers PA Mburu RG Kato CM Rhodes SM. Assessment of planning target volume margins for intensity-modulated radiotherapy of the prostate gland: role of daily inter- and intrafraction motion. Int J Radiat Oncol Biol Phys. (2010) 78:1579–85. 10.1016/j.ijrobp.2010.02.001 20472357

[29] Wust P Joswig M Graf R Böhmer D Beck M Barelkowski T. Dosimetric implications of inter- and intrafractional prostate positioning errors during tomotherapy: comparison of gold marker-based registrations with native MVCT. Strahlenther Onkol. (2017) 193:700–6. 10.1007/s00066-017-1141-x 28466155

[30] Beltran C Herman MG Davis BJ. Planning target margin calculations for prostate radiotherapy based on intrafraction and interfraction motion using four localization methods. Int J Radiat Oncol Biol Phys. (2008) 70:289–95. 10.1016/j.ijrobp.2007.08.040 17919837

[31] Shelley LEA Scaife JE Romanchikova M Harrison K Forman JR Bates AM. Delivered dose can be a better predictor of rectal toxicity than planned dose in prostate radiotherapy. Radiother Oncol. (2017) 123:466–71. 10.1016/j.radonc.2017.04.008 28460825 PMC5486775

[32] Kubota Y Okamoto M Li Y Shiba S Okazaki S Komatsu S. Evaluation of intensity- and contour-based deformable image registration accuracy in pancreatic cancer patients. Cancers. (2019) 11:1447. 10.3390/cancers11101447 31569617 PMC6826682

